# Quasi-continuous metasurface for ultra-broadband and polarization-controlled electromagnetic beam deflection

**DOI:** 10.1038/srep17733

**Published:** 2015-12-04

**Authors:** Yanqin Wang, Mingbo Pu, Zuojun Zhang, Xiong Li, Xiaoliang Ma, Zeyu Zhao, Xiangang Luo

**Affiliations:** 1 State Key Laboratory of Optical Technologies on Nano-Fabrication and Micro-Engineering, Institute of Optics and Electronics, Chinese Academy of Science, P.O. Box 350, Chengdu 610209, China; 2 Key Laboratory of Optoelectronic Technology and System, Ministry of Education, Chongqing University, Chongqing 400030, China

## Abstract

Two-dimensional metasurface has attracted growing interest in recent years, owing to its ability in manipulating the phase, amplitude and polarization state of electromagnetic wave within a single interface. However, most existing metasurfaces rely on the collective responses of a set of discrete meta-atoms to perform various functionalities. In this paper, we presented a quasi-continuous metasurface for high-efficiency and broadband beam steering in the microwave regime. It is demonstrated both in simulation and experiment that the incident beam deviates from the normal direction after transmitting through the ultrathin metasurface. The efficiency of the proposed metasurface approximates to the theoretical limit of the single-layer metasurface in a broad frequency range, owing to the elimination of the circuit resonance in traditional discrete structures. The proposed scheme promises potential applications in broadband electromagnetic modulation and communication systems, etc.

Manipulation of electromagnetic wave based on gradient phase distribution has found numerous applications, such as beam splitting, focusing and imaging. Conventional methods achieve the control of the phase by means of subtly designing the geometries and refractive index profile so that different phase accumulates along the transmission path^1^,^2^. This approach was extensively exploited to engineer a variety of optical elements such as optical lens, wave plates, spiral phase plate as well as holograms^3^,^4^,^5^,^6^. However, as the refractive index available in naturally occurring materials is typically small, a large thickness compared to the wavelength is required for conventional components^2^, which brings significant limitations to the integration of microwave and optical systems.

Metasurfaces, as two-dimensional (2D) artificially structured materials, have attracted significant interest due to the ultrathin profiles and their unique electromagnetic properties. Numerous exotic phenomena such as flat lensing, ultra-broadband absorption, and spin-Hall effect of light have been demonstrated very recently^7^,^8^,^9^,^10^,^11^,^12^,^13^,^14^,^15^,^16^,^17^,^18^,^19^,^20^,^21^. In particular, benefiting from the local abrupt phase retardations, full control of phase profile of light over the subwavelength scale was achieved^1^,^22^,^23^,^24^. In traditional plasmonic metasurface, the phase retardation is highly dependent on the light wavelength, because the resonant interaction has been utilized^6^,^22^,^25^. To obtain dispersionless phase modulation, the phase shift originating from polarization conversion in anisotropic elements are widely exploited, either for the linear or circular polarization^1^,^8^. Although this kind of phase shift provides much more flexibility to the electromagnetic wave manipulation, there are still some problems should be addressed before their practical applications. For example, most current metasurfaces relied on the combination of different (disconnected) discrete elements to create the phase gradient, while each individual element (meta-atom) introduced only a locally constant phase. Such discrete design may be disadvantageous in applications where high diffraction efficiency is required^26^,^27^. Furthermore, the scattering efficiency for discrete metasurface is only large enough for a particular spectral region, owing to the electromagnetic resonance^28^.

Here, a quasi-continuous element is proposed to improve the cross-polarization conversion efficiency and the bandwidth of metasurface under circular polarized illumination. A power efficiency close to 25%, which is predicted as a theoretical limit for the single-layer metasurface^21^, is realized in frequencies ranging from 10 to 20 GHz. Instead of periodically adjusting the orientation of the metallic dipole antennas, a phase gradient covering [0, 360°] is realized in a single quasi-continuous element. With this quasi-continuous structure, we numerically design a broadband microwave deflector for circular polarized incidence. The experimental results, in excellent agreement with theoretical analysis and simulations, show that the incident beam is deflected when it propagates through the interface of the quasi-continuous metasurface, whereas the refraction angle is determined by the handedness and frequency of the incidence beam.

## Results

### Theoretical model and structure design

**Figure 1:**
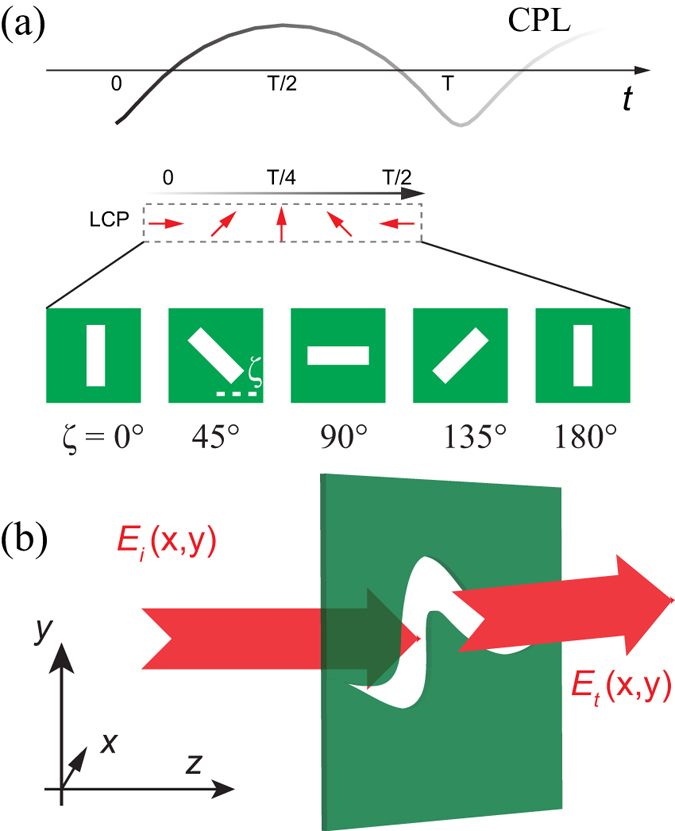
Schematic of the abnormal phase gradient induced by a curved slit in a metallic screen. (**a**) Physical mechanism of the geometric phase in space-variant subwavelength apertures. The angle between the main axis of the anisotropic aperture and the instantaneous polarization state of the CPL determines the phase shift. (**b**) The phase of electromagnetic waves is changed via a curved subwavelength slit, which could be approximated as composed of space-variant anisotropic apertures. Here *E*_*i*_ and *E*_*t*_ denote the incident and transmitted electric fields.

It is well known that the electric field vector is rotating in a circularly polarized light (CPL). As a consequence, a polarization rotation process is physically identical to a phase retardation in this particular case. As shown in Fig. 1a, when a CPL illuminates on an array made of space-variant anisotropic slits, the polarization as well as the phase shift of the transmitted light will be different. Considering that the subwavelength slit has an orientation angle of *ζ* with respect to the *x*-axis, the output fields can be written as a combination of phase retardation and polarization rotation:


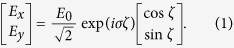


While the item exp(*iσζ*) stems from the phase retardation induced by the polarization rotation of CPL, the matrix in the right hand denotes the polarization selection process of the anisotropic slit. By some mathematical manipulation, the complex fields could be re-written as a combination of two circularly polarized light:


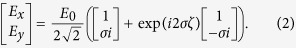


Obviously, the output fields are composed by two counter-rotating CPL, where the cross-polarized light has an additional phase shift of 2*σζ*. Here *σ* = ±1 denotes the left-handed circular polarization (LCP) and right-handed circular polarization (RCP), respectively.

According to the metasurface-assisted law of refraction and reflection (MLRR)^1^,^2^,^24^,^29^, a gradient phase in the metasurface could make the input light be deflected to the pre-defined direction. In order to realize a full control of the wavefront, many works have been devoted to design subwavelength antennas array with space-variant orientations. However, these designs suffer from the relatively small bandwidth owing to the resonant nature. Recently, we proposed a semi-continuous catenary structure and demonstrated that it could be used as a perfect phase modulator in the optical frequencies^28^. As illustrated in Fig. 1b, a properly designed curved slit could be used to control the phase front of the cross-polarized transmission. Nevertheless, in the previous results, there is still observable oscillations owing to the plasmonic resonance. It was predicted that these resonances could be eliminated by shifting the operating frequency to lower frequency regime, where the metal could be approximated as perfect electric conductor (PEC). The catenary structure is obtained by connecting two catenary curves with a vertical shift of Δ^28^.


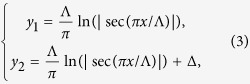


It should be noted that the catenary structure has a non-uniform width, which is an inevitable result of the topologic properties. Since the inclination angle of the catenary aperture has a form of:


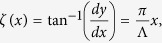


there is a linear phase distribution of Φ(*x*) = 2*σπx*/Λ at the output boundary of the catenary aperture. Thus the phase shift of a single catenary aperture could cover [0, 2π], implying that the catenary structure could be utilized as a unique building block of phase-type metasurface. As shown by the MLRR, the deflection angle could be evaluated as *θ* = *σ*arcsin(*λ*/Λ), where *λ* is the wavelength of the incident light. When *λ* is larger than Λ, the deflection angle becomes imaginary, consequently the incident light would be converted to evanescent surface wave^30^.

**Figure 2:**
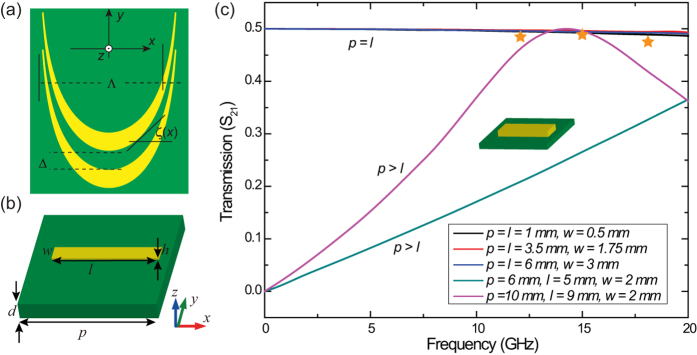
Design of the quasi-continuous metasurface made from catenary structures. (**a**) Front-view of the catenary structure. (**b**) Schematic of the metallic rod which was considered as the subunit of the quasi-continuous metasurface. (**c**) Numerically simulated conversion efficiency for different geometric parameters. The star symbols represent the results evaluated from the full-model given in Fig. (3).

In the microwave, terahertz and far infrared regimes, metals such as copper, gold, and aluminum could be treated as near PEC in many non-resonant metamaterials. As a result, catenaries in these frequency bands could be treated as true broadband structures. As shown in Fig. 2a,b, the unit cell of the catenary could be approximated as continuous gratings with space-variant orientations. In the numerical evaluations, the grating has variant geometric parameters *p, l* and *w*. For continuous grating, we have *p* = *l* and *w* = Δ, which is the width and the shift of the catenary structure. The material for metal is chosen as Copper with conductivity of 5.7 × 10^7^ S/m. The dielectric substrate is chosen as Rogers 3003 with dielectric constant of *ε* = 3 and thickness of *d* = 0.5 mm. The thickness of the copper is *h* = 0.017 mm. Figure 2c depicts the amplitude of the conversion efficiency for CPL transmitted to its cross-polarization (LCP-to-RCP, or RCP-to-LCP). Interestingly, we noted that the conversion efficiency is nearly a constant for the continuous gating (*p* = *l*)^31^, which is almost independent of the width *w*. In contrary, when *l* < *p*, the conversion efficiency only reaches its maximum in the frequency band near the resonance. The maximal amplitude of the cross-polarized wave is close to 0.5, coinciding with a power efficiency of 25%^32^,^33^.

### Simulation and experiment results

**Figure 3:**
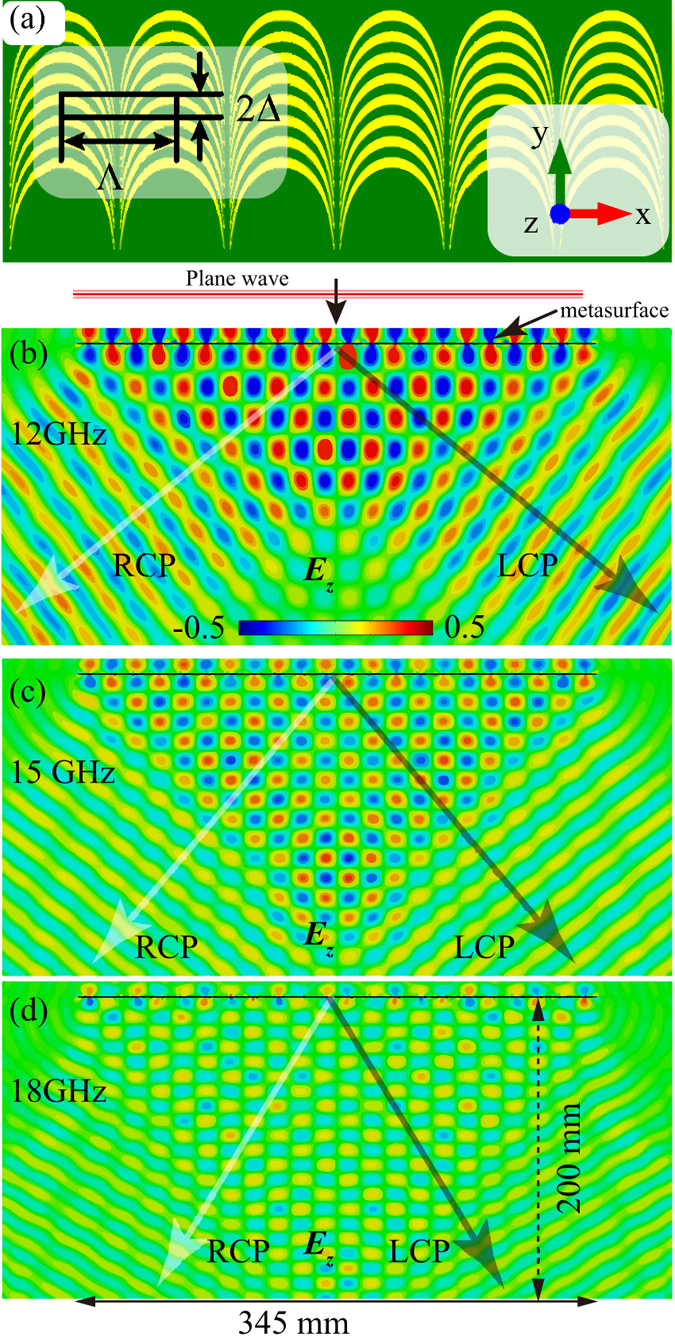
Numerical simulation of the quasi-continuous metasurface deflector. (**a**) Schematic of the full-model of the catenary-based deflector. (**b–d**) The *z*-component of the transmitted electric field (*E*_*z*_) distributed in the *xz*-plane for linear polarization incidence (1 V/m) at 12, 15 and 18 GHz. The directions of the LCP and RCP components are indicated.

To verify our proposed scheme, we investigate the deflection phenomenon of the quasi-continuous metasurface for both RCP and LCP under the normal incidence. In the following, we demonstrate that a linear array of catenary could serve as a broadband polarization-dependent beam deflector. The schematic diagram of deflector is shown as Fig. 3a. The quasi-continuous metasurface has a periodicity of Λ = 31.4 mm in the *x*-direction and 2Δ = 6 mm in the *y*-direction, respectively.

We simulated the performance of the catenary array with commercial software CST MWS. The *z*-component of the transmitted electric field (*E*_*z*_) for normal incidence at three frequencies of 12, 15 and 18 GHz are plotted in Fig. 3b–d. Since linearly polarized incidence was adopted, the fields comprise both LCP and RCP components, with opposite deflecting directions as indicated in each panel. It could be seen that the deflection angles for 12, 15 and 18 GHz are about 50°, 40° and 30°, agreeing well with the theoretical values 52.79°, 39.58° and 32.07°, as given by *θ* = *σ*arcsin(*λ*/Λ). Since the amplitude of the incident electric field is set as 1 V/m, the theoretical maxima of *E*_z_ should be 0.35λ/Λ, corresponding to 0.28, 0.22, 0.18 V/m at frequencies of 12, 15 and 18 GHz. As indicated by the star symbols in Fig. 2(c), the conversion efficiency of the sample can be evaluated by comparing Ez with the theoretical evaluations, presenting a good match with the results shown in Fig. 2(c).

**Figure 4:**
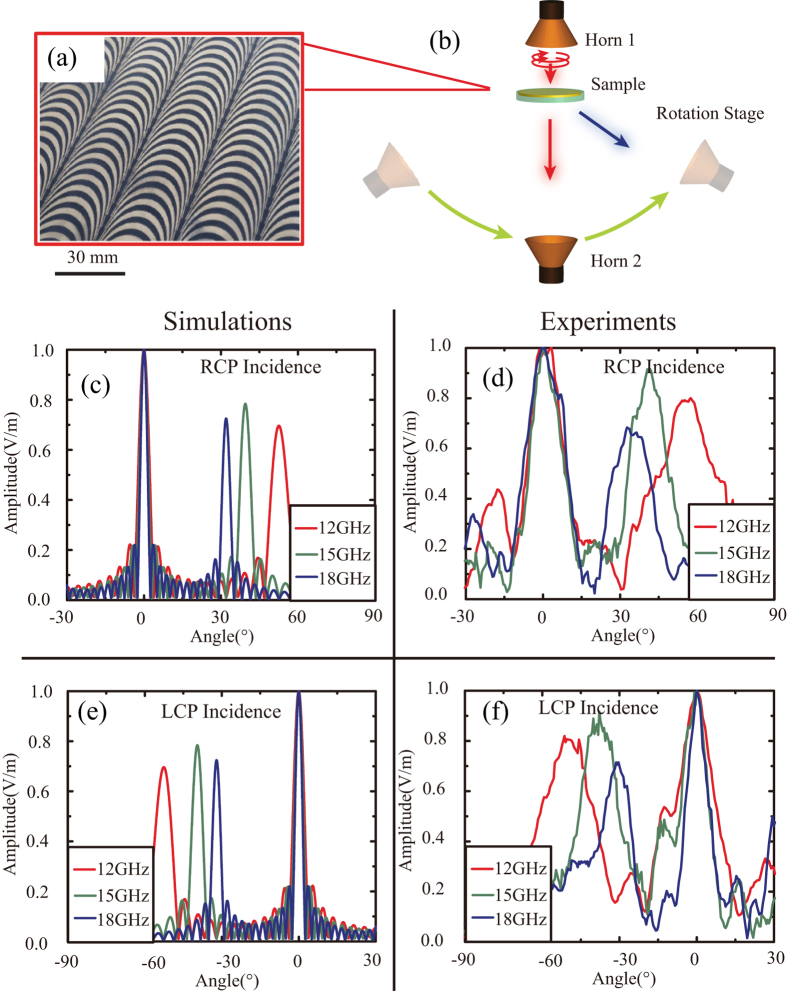
Experimental verification of the catenary-based deflector. (**a**) Optical photograph of the fabricated metasurface deflector. Scale bar: 30 mm. (**b**) Schematic diagram of the experiment setup. (**c–f**) Numerical and experimental results of the overall transmitted electromagnetic wave in the far-fields with RCP (**c** and **d**) and LCP (**e**,**f**) incidence at 12, 15, and 18 GHz.

The designed catenary array was fabricated on a 17 μm-thick copper film patterned on a 0.5 mm thick Rogers 3003 substrate by standard printed circuit board (PCB) technology (Fig. 4a). The electromagnetic wave scattered by the catenary array was measured using an R&S ZVA40 vector network analyzer in the microwave anechoic chamber. A schematic diagram of the measurement is shown in Fig. 4b. The transmitting horn antenna (horn 1) is circularly polarized to achieve circular polarization illumination. The receiving horn antenna (horn 2) is linearly polarized, thus both the co-polarized and cross-polarized transmission could be received. Figure 4c,e depicts the simulated results of the normalized intensity in the far-field, giving us a clear understand about the changing of the deflection angle with frequency and handedness. The co-polarized component lies at the 0° direction, whereas the cross-polarized component is deflected into an anomalous refraction angle.

The experimental deflection effect with RCP and LCP incidence is shown in Fig. 4d,f, respectively. For RCP incidence, the tested deflection angles are 52.83°, 40.62° and 32.69° for *f* = 12, 15 and 18 GHz, respectively, agreeing well with the theoretical deflection angles as shown in Fig. 4c. Figure 4f shows that the angular spectra for LCP incidence, where the deflection angles are −51.36°, −38.99° and −31.28° for *f* = 12, 15 and 18 GHz, respectively. The measured beam width is broader than the simulation results because the gain of the horn adopted in the measurement system is small thus the radiated beam has a relatively large beam width. Nevertheless, the ratios between the maxima of the cross-polarization and co-polarization are close to or even larger than that given by the full-model simulations.

## Discussions

In summary, we have proposed a quasi-continuous metasurface to realize the phase modulation of electromagnetic wave. The novel design is characterized by high cross-polarization conversion efficiency within a broadband range covering almost all of the electromagnetic spectrum. This is similar to the ultrabroadband frequency responses (absorption^34^,^35^ and polarization conversion^31^) given in previous results.

The sample was tested on a vector network analyzer, with deflection angle agreeing well with the theoretical prediction. Such a design can be also extended to the terahertz-frequency, infrared and visible light regime. The beam control technology based on phase discontinuities in continuous metasurfaces may find more potential applications, such as imaging, optical manipulation and so on.

## Methods

### Measurements

The experimental measurement was carried out on a vector network analyzer (R&S ZVA40) in a microwave anechoic chamber. Two standard horn antennas were used as transmitter (circularly polarized) and receiver (linearly polarized), and connected to the two ports of the vector network analyzer. The receiver was mounted on a turntable to scan the transmitted electric field from the metasurface.

## Additional Information

**How to cite this article**: Wang, Y. *et al.* Quasi-continuous metasurface for ultra-broadband and polarization-controlled electromagnetic beam deflection. *Sci. Rep.* **5**, 17733; doi: 10.1038/srep17733 (2015).

## References

[b1] Yu N. *et al.* Light propagation with phase discontinuities: generalized laws of reflection and refraction. Science 334, 333–337 (2011). 21885733 10.1126/science.1210713

[b2] Luo X. Principles of electromagnetic waves in metasurfaces. Sci. China-Phys. Mech. Astron. 58, 594201 (2015).

[b3] Wang D., Gu Y., Gong Y., Qiu C.-W. & Hong M. An ultrathin terahertz quarter-wave plate using planar babinet-inverted metasurface. Opt. Express 23, 11114–11122 (2015). 25969207 10.1364/OE.23.011114

[b4] Beijersbergen M. W., Coerwinkel R. P. C., Kristensen M. & Woerdman J. P. Helical-wave-front laser-beams produced with a spiral phase plate. Opt. Commun. 112, 321–327 (1994).

[b5] Larouche S., Tsai Y.-J., Tyler T., Jokerst N. M. & Smith D. R. Infrared metamaterial phase holograms. Nat. Mater. 11, 450–454 (2012). 22426458 10.1038/nmat3278

[b6] Luo X. & Yan L. Surface plasmon polaritons and its applications. IEEE Photonics J. 4, 590–595 (2012).

[b7] Pendry J. B. Negative refraction makes a perfect lens. Phys. Rev. Lett. 85, 3966–3969 (2000). 11041972 10.1103/PhysRevLett.85.3966

[b8] Hasman E., Kleiner V., Biener G. & Niv A. Polarization dependent focusing lens by use of quantized Pancharatnam–Berry phase diffractive optics. Appl. Phys. Lett. 82, 328–330 (2003).

[b9] Luo X. & Ishihara T. Surface plasmon resonant interference nanolithography technique. Appl. Phys. Lett. 84, 4780–4782 (2004).

[b10] Hao J. *et al.* Manipulating electromagnetic wave polarizations by anisotropic metamaterials. Phys. Rev. Lett. 99, 063908 (2007). 17930829 10.1103/PhysRevLett.99.063908

[b11] Feng Q., Pu M., Hu C. & Luo X. Engineering the dispersion of metamaterial surface for broadband infrared absorption. Opt. Lett. 37, 2133–2135 (2012). 22660145 10.1364/OL.37.002133

[b12] Aieta F. *et al.* Aberration-free ultra-thin flat lenses and axicons at telecom wavelengths based on plasmonic metasurfaces. Nano Lett. 12, 4932–4936 (2012). 22894542 10.1021/nl302516v

[b13] Yin X., Ye Z., Rho J., Wang Y. & Zhang X. Photonic spin Hall effect at metasurfaces. Science 339, 1405–1407 (2013). 23520105 10.1126/science.1231758

[b14] Pu M. *et al.* Anisotropic meta-mirror for achromatic electromagnetic polarization manipulation. Appl. Phys. Lett. 102, 131906 (2013).

[b15] Pan W. *et al.* A beam steering horn antenna using active frequency selective surface. IEEE Trans. Antennas Propag. 61, 6218–6223 (2013).

[b16] Ling X. *et al.* Giant photonic spin Hall effect in momentum space in a structured metamaterial with spatially varying birefringence. Light Sci. Appl. 4, e290 (2015).

[b17] Ma X. *et al.* An active metamaterial for polarization manipulating. Adv. Opt. Mater. 2, 945–949 (2014).

[b18] Lin D., Fan P., Hasman E. & Brongersma M. L. Dielectric gradient metasurface optical elements. Science 345, 298–302 (2014). 25035488 10.1126/science.1253213

[b19] Aieta F., Kats M. A., Genevet P. & Capasso F. Multiwavelength achromatic metasurfaces by dispersive phase compensation. Science 347, 1342–1345 (2015). 25700175 10.1126/science.aaa2494

[b20] Zheng G., Mühlenbernd H., Kenney M., Li G. & Zhang S. Metasurface holograms reaching 80% efficiency. Nat. Nanotechnol. 10, 308–312 (2015). 25705870 10.1038/nnano.2015.2

[b21] Ding X. *et al.* Ultrathin Pancharatnam–Berry metasurface with maximal cross-polarization efficiency. Adv. Mater. 27, 1195–1200 (2015). 25545285 10.1002/adma.201405047

[b22] Verslegers L. *et al.* Planar lenses based on nanoscale slit arrays in a metallic film. Nano Lett. 9, 235–238 (2008). 19053795 10.1021/nl802830y

[b23] Luo X., Pu M., Ma X. & Li X. Taming the electromagnetic boundaries via metasurfaces: from theory and fabrication to functional devices. Int. J. Antennas Propag. 2015, 204127 (2015).

[b24] Pu M. *et al.* Spatially and spectrally engineered spin-orbit interaction for achromatic virtual shaping. Sci. Rep. 5, 9822 (2015). 25959663 10.1038/srep09822 PMC4426594

[b25] Sun S. *et al.* Gradient-index meta-surfaces as a bridge linking propagating waves and surface waves. Nat. Mater. 11, 426–431 (2012). 22466746 10.1038/nmat3292

[b26] Li Z., Palacios E., Butun S. & Aydin K. Visible-frequency metasurfaces for broadband anomalous reflection and high-efficiency spectrum splitting. Nano Lett. 15, 1615–1621 (2015). 25664815 10.1021/nl5041572

[b27] Zhang L. *et al.* Anomalous behavior of nearly-entire visible band manipulated with degenerated image dipole array. Nanoscale 6, 12303–12309 (2014). 25163776 10.1039/c4nr03163f

[b28] Pu M. *et al.* Catenary optics for achromatic generation of perfect optical angular momentum. Sci. Adv. 1, e1500396 (2015). 26601283 10.1126/sciadv.1500396 PMC4646797

[b29] Pu M. *et al.* Broadband anomalous reflection based on low-Q gradient meta-surface. AIP Adv. 3, 052136 (2013).

[b30] Lin J. *et al.* Polarization-controlled tunable directional coupling of surface plasmon polaritons. Science 340, 331–334 (2013). 23599488 10.1126/science.1233746

[b31] Wang Y. *et al.* Dynamic manipulation of polarization states using anisotropic meta-surface. Opt. Commun. 319, 14–16 (2014).

[b32] Chen X. *et al.* Dual-polarity plasmonic metalens for visible light. Nat. Commun. 3, 1198 (2012). 23149743 10.1038/ncomms2207 PMC3514495

[b33] Ma X. *et al.* A planar chiral meta-surface for optical vortex generation and focusing. Sci. Rep. 5, 10365 (2015). 25988213 10.1038/srep10365 PMC4437373

[b34] Pu M. *et al.* Ultrathin broadband nearly perfect absorber with symmetrical coherent illumination. Opt. Express 20, 2246–2254 (2012). 22330464 10.1364/OE.20.002246

[b35] Li S. *et al.* Broadband perfect absorption of ultrathin conductive films with coherent illumination: Superabsorption of microwave radiation. Phys. Rev. B 91, 220301 (2015).

